# Supplementary material for: Cytochrome P450 Enzymes and Drug Metabolism in Humans
Source: Int J Mol Sci. 2021 Nov 26;22(23):12808. doi: 10.3390/ijms222312808 (PMC8657965; doi:10.3390/ijms222312808)
Supplement: Supplementary file 1 [file ijms-22-12808-s001.zip › ijms-1450839-supplementary.pdf]

Supplementary Table S1. Pharmacology of major drug-metabolizing CYPs in humans.

CYPs	Tissue sites	Substrates			Inhibitors	Inducers
CYP1A2 [1-3]	Liver	amitriptyline<br>propranolol<br>cyclobenzaprine<br>tacrine<br>haloperidol<br>warfarin<br>naproxen	phenacetin<br>clomipramine<br>ropivacaine<br>estradiol<br>verapamil<br>mexiletine<br>zolmitriptan	caffeine<br>riluzole<br>fluvoxamine<br>theophylline<br>imipramine<br>zileuton<br>ondansetron	amiodarone<br>cimetidine<br>fluoroquinolones<br>fluvoxamine<br>furafylline<br>interferon<br>methoxsalen<br>mibefradil<br>ticlopidine	broccoli<br>brussel sprouts<br>char-grilled meat<br>insulin<br>methyl cholanthrene<br>modafinil<br>α-naphthoflavone<br>omeprazole<br>tobacco
CYP2B6 [2, 4, 5]	Liver, lung	bupropion	cyclophosphamide	ifosphamide	thiotepa	phenobarbital<br>phenytoin<br>rifampin
CYP2C19 [2, 6]	Liver	amitriptyline<br>pantoprazole<br>cyclophosphamide<br>E-3810<br>proguanil<br>indomethacin<br>teniposide<br>moclobemide	omeprazole<br>clomipramine<br>phenobarbitone<br>progesterone<br>imipramine N-deME<br>R-warfarin<br>S-mephenytoin<br>nelfinavir	citalopram<br>phenytoin<br>diazepam<br>hexobarbital<br>propranolol<br>lansoprazole<br>R-mephobarbital<br>nilutamide	cimetidine<br>felbamate<br>fluoxetine<br>fluvoxamine<br>indomethacin<br>ketoconazole<br>lansoprazole<br>modafinil<br>omeprazole<br>paroxetine<br>probenicid<br>ticlopidine<br>topiramate	carbamazepine<br>norethindrone<br>prednisone<br>rifampin
CYP2C9 [7, 8]	Liver	amitriptyline<br>piroxicam<br>fluoxetine<br>suprofen	phenytoin<br>diclofenac<br>sulfamethoxazole<br>glipizide	celecoxib<br>rosiglitazone<br>fluvastatin<br>tamoxifen	amiodarone<br>fluconazol<br>fluvastatin<br>fluvoxamine	rifampin<br>secobarbital

Supplementary Table S1 contd.......

CYPs	Tissue sites	Substrates			Inhibitors	Inducers
		ibuprofen	torsemide	irbesartan	isoniazid	
		tolbutamide	losartan	S-warfarin	lovastatin	
		naproxen			paroxetine	
					phenylbutazone	
					probenicid	
					sertraline	
					sulfaphenazole	
					teniposide	
					trimethoprim	
					zafirlukast	
CYP2D6 [2, 9]	Liver	alprenolol	S-metoprolol	amitriptyline	amiodarone	dexamethasone
		mexiletine	amphetamine	minaprine	celecoxib	rifampin
		bufuralol	nortriptyline	carvedilol	chlorpheniramine	
		ondansetron	chlorpromazine	paroxetine	chlorpromazine	
		clomipraminecodeine	perhexiline	perphenazine	cimetidine	
		debrisoquine	phenacetin	desipramine	clomipramine	
		phenformin	dexfenfluramine	propafenone	cocaine	
		dextromethorphan	propanolol	encainide	doxorubicin	
		quanoxan	flecainide	risperidone	fluoxetine	
		fluoxetine	sparteine	fluvoxamine	halofantrine	
		tamoxifen	haloperidol	thioridazine	levomepromazine	
		imipramine	timolol	lidocaine	methadone	
		tramadol	metoclopramide	venlafaxine	moclobemide	
		methoxyamphetamine			paroxetine	
					quinidine	
					ranitidine	
					haloperidol	
					ritonavir	
					sertraline	

Supplementary Table S1 contd.......

CYPs	Tissue sites	Substrates			Inhibitors	Inducers
		acetaminophen	halothane	aniline	diethyl-dithiocarbamate	ethanol
CYP2E1	Liver	isoflurane	benzene	methoxyflurane	disulfiram	isoniazid
[10, 11]		chlorzoxazone	N,N-dimethylformamide	enflurane		
		sevoflurane	ethanol	theophylline		
		alfentanyl	lovastatin	codeine	amiodarone	barbiturates
		alprazolam	methadone	ritonavir	azithromycin	carbamazepine
		amlodipine	midazolam	cyclosporine	cimetidine	efavirenz
		astemizole	nelfinavir	salmeterol	ciprofloxacin	glucocorticoids
		atorvastatin	nifedipine	dapsone	clarithromycin	modafinil
		azithromycin	nisoldipine	saquinavir	delaviridine	nevirapine
		buspirone	nitrendipine	dextromethorphan	diethyl-dithiocarbamate	phenobarbital
		caffeine	odanestron	sildenafil	diltiazem	phenytoin
		cerivastatin	pimozide	diazepam	erythromycin	pioglitazone
		chlorpheniramine	progesterone	simvastatin	fluconazole	rifampin
CYP3A4	Liver	cisapride	propranolol	diltiazem	fluvoxamine	troglitazone
CYP3A5	Liver, lung	clarithromycin	quinidine	tacrolimus	gestodene	
CYP3A7	Fetal liver	cocaine	quinine	erythromycin	grapefruit juice	
[2, 12]		tamoxifen	estradiol	taxol	indinavir	
		felodipine	terfenadine	fentanyl	interleukin-10	
		testosterone	finasteride	trazodone	itraconazole	
		haloperidol	triazolam	hydrocortisone	ketoconazole	
		verapamil	indinavir	vincristine	mibefradil	
		lercanidipine	zaleplon	lidocaine	mifepristone	
		zolpidem			nefazodone	
					nelfinavir	
					norfloxacin	
					ritonavir	
					norfluoxetine	

References

1. Omiecinski, C. J.; Redlich, C. A.; Costa, P., Induction and developmental expression of cytochrome P450IA1 messenger RNA in rat and human tissues: detection by the polymerase chain reaction. *Cancer Res* **1990,** *50* (14), 4315-21.

2. Rendic, S., Summary of information on human CYP enzymes: human P450 metabolism data. *Drug Metab Rev* **2002,** *34* (1-2), 83-448.

3. Racha, J. K.; Rettie, A. E.; Kunze, K. L., Mechanism-based inactivation of human cytochrome P450 1A2 by furafylline: detection of a 1:1 adduct to protein and evidence for the formation of a novel imidazomethide intermediate. *Biochemistry* **1998,** *37* (20), 7407-19.

4. Code, E. L.; Crespi, C. L.; Penman, B. W.; Gonzalez, F. J.; Chang, T. K.; Waxman, D. J., Human cytochrome P4502B6: interindividual hepatic expression, substrate specificity, and role in procarcinogen activation. *Drug Metab Dispos* **1997,** *25* (8), 985-93.

5. Zanger, U. M.; Klein, K.; Saussele, T.; Blievernicht, J.; Hofmann, M. H.; Schwab, M., Polymorphic CYP2B6: molecular mechanisms and emerging clinical significance. *Pharmacogenomics* **2007,** *8* (7), 743-59.

6. Niwa, T.; Yamazaki, H., Comparison of cytochrome P450 2C subfamily members in terms of drug oxidation rates and substrate inhibition. *Curr Drug Metab* **2012,** *13* (8), 1145-59.

7. Miners, J. O.; Birkett, D. J., Cytochrome P4502C9: an enzyme of major importance in human drug metabolism. *Br J Clin Pharmacol* **1998,** *45* (6), 525-38.

8. Mancy, A.; Dijols, S.; Poli, S.; Guengerich, P.; Mansuy, D., Interaction of sulfaphenazole derivatives with human liver cytochromes P450 2C: molecular origin of the specific inhibitory effects of sulfaphenazole on CYP 2C9 and consequences for the substrate binding site topology of CYP 2C9. *Biochemistry* **1996,** *35* (50), 16205-12.

9. Kawata, T.; Tokunaga, S.; Murai, M.; Masuda, N.; Haruyama, W.; Shoukei, Y.; Hisada, Y.; Yanagida, T.; Miyazaki, H.; Wada, M.; Akizawa, T.; Fukagawa, M., A novel calcimimetic agent, evocalcet (MT-4580/KHK7580), suppresses the parathyroid cell function with little effect on the gastrointestinal tract or CYP isozymes in vivo and in vitro. *PLoS One* **2018,** *13* (4), e0195316.

10. Shrivas, K.; Mindaye, S. T.; Getie-Kebtie, M.; Alterman, M. A., Mass spectrometry-based proteomic analysis of human liver cytochrome(s) P450. *Toxicol Appl Pharmacol* **2013,** *267* (1), 125-36.

11. Yoo, J. S.; Cheung, R. J.; Patten, C. J.; Wade, D.; Yang, C. S., Nature of N-nitrosodimethylamine demethylase and its inhibitors. *Cancer Res* **1987,** *47* (13), 3378-83.

12. Shimada, T.; Yamazaki, H.; Mimura, M.; Inui, Y.; Guengerich, F. P., Interindividual variations in human liver cytochrome P-450 enzymes involved in the oxidation of drugs, carcinogens and toxic chemicals: studies with liver microsomes of 30 Japanese and 30 Caucasians. *J Pharmacol Exp Ther* **1994,** *270* (1), 414-23.
